# The Unity of Redox and Structural Remodeling of Brown Adipose Tissue in Hypothyroidism

**DOI:** 10.3390/antiox10040591

**Published:** 2021-04-12

**Authors:** Marija Aleksic, Andjelika Kalezic, Luciano Saso, Aleksandra Jankovic, Bato Korac, Aleksandra Korac

**Affiliations:** 1 Center for Electron Microscopy, Faculty of Biology, University of Belgrade, 11000 Belgrade, Serbia; marija.aleksic@bio.bg.ac.rs (M.A.); koracb@ibiss.bg.ac.rs (B.K.); 2 Institute for Biological Research “Sinisa Stankovic”—National Institute of Republic of Serbia, University of Belgrade, 11000 Belgrade, Serbia; andjelika.kalezic@ibiss.bg.ac.rs; 3 Department of Physiology and Pharmacology “Vittorio Erspamer”, Faculty of Pharmacy and Medicine, Sapienza University of Rome, 00161 Rome, Italy; luciano.saso@uniroma1.it

**Keywords:** hypothyroidism, brown adipose tissue, antioxidant defense compartmentalization, mitochondria/peroxisome/lipid body unit

## Abstract

Brown adipose tissue (BAT) is important for maintaining whole-body metabolic and energy homeostasis. However, the effects of hypothyroidism, one of the most common diseases worldwide, which increases the risk of several metabolic disorders, on BAT redox and metabolic homeostasis remain mostly unknown. We aimed to investigate the dynamics of protein expression, enzyme activity, and localization of antioxidant defense (AD) enzymes in rat interscapular BAT upon induction of hypothyroidism by antithyroid drug methimazole for 7, 15, and 21 days. Our results showed an increased protein expression of CuZn- and Mn-superoxide dismutase, catalase, glutamyl–cysteine ligase, thioredoxin, total glutathione content, and activity of catalase and thioredoxin reductase in hypothyroid rats, compared to euthyroid control. Concomitant with the increase in AD, newly established nuclear, mitochondrial, and peroxisomal localization of AD enzymes was found. Hypothyroidism also potentiated associations between mitochondria, peroxisomes, and lipid bodies, creating specific structural–functional units. Moreover, hypothyroidism induced protein expression and nuclear translocation of a master regulator of redox-metabolic homeostasis, nuclear factor erythroid 2-related factor 2 (Nrf2), and an increased amount of 4-hydroxynonenal (4-HNE) protein adducts. The results indicate that spatiotemporal overlap in the remodeling of AD is orchestrated by Nrf2, implicating the role of 4-HNE in this process and suggesting the potential mechanism of redox-structural remodeling during BAT adaptation in hypothyroidism.

## 1. Introduction

Hypothyroidism is one of the most common diseases worldwide, which affects up to 5% of the general population, with a further estimated 5% being undiagnosed [1]. Moreover, hypothyroidism is a risk factor for developing a wide range of metabolic diseases such as obesity, metabolic syndrome, non-alcoholic fatty liver disease, insulin resistance, and type 2 diabetes [2,3,4,5]. A large body of evidence shows that disturbed redox homeostasis is an essential part of the etiology and pathophysiology of complex metabolic diseases, including thyroid diseases [6,7,8,9,10,11,12]. Reactive oxygen and nitrogen species (ROS and RNS) regulate every aspect of oxidative metabolism and metabolic regulation [13,14]. Redox-sensitive transcription factors, such as nuclear factor erythroid 2-related factor 2 (Nrf2), play a pivotal role in maintaining redox homeostasis and integrating redox and metabolic regulatory networks by regulating the expression of genes involved in the antioxidant and detoxifying response, metabolic regulation, and mitochondrial function [15,16,17,18]. As a result, there is a growing research interest in identifying redox biomarkers and redox-sensitive targets for diagnosing and treating metabolic diseases [19,20,21].

Brown adipose tissue (BAT) is a crucial site for regulating overall energy homeostasis. It is a highly specialized organ that uses the energy stored in the form of lipids to produce heat through nonshivering thermogenesis and is the most pronounced in small rodents and newborn mammals [22]. Recent data show that BAT plays an important role in the regulation of whole-body energy metabolism in adult humans as well as that metabolic diseases, including obesity, type 2 diabetes, and insulin resistance, could be controlled by modulating the activity of BAT [23,24,25,26,27]. All essential aspects of BAT function are regulated by thyroid hormones, including the regulation of gene expression, availability of nutrients, lipogenesis, lipolysis, thermogenesis, biogenesis of mitochondria and peroxisomes, and antioxidant defense (AD) [28,29,30,31]. It is well-known that hypothyroid rats acclimated to the cold display cold-intolerance to different degrees [32,33,34,35]. However, accumulating evidence indicates that in rats acclimated to room temperature, hypothyroidism partially mimics the effects of chronic exposure to cold (cold-induced thermogenesis) in BAT [28,32,33]. Such a thermogenic activation of BAT is based on extensive redox remodeling, which allows for the increase in metabolic activity [36,37,38,39]. Namely, activation of BAT undoubtedly leads to ROS and RNS production, which subsequently participate in numerous redox-sensitive signaling pathways involved in tissue blood perfusion, capillary remodeling, brown adipocyte proliferation and differentiation, apoptosis, and mitochondriogenesis [40,41,42,43,44,45]. In response to increased metabolic demands and reactive species signaling, AD sustains redox homeostasis and maintains tissue structural and functional integrity. Nevertheless, there is almost no data on the dynamics of underlying changes to the redox regulation in BAT following metabolic and structural remodeling prompted by hypothyroid conditions. Bearing that in mind, we decided to use methimazole-induced hypothyroidism as an experimental model of organellar remodeling in BAT.

Here, we aimed to examine changes to the redox regulation in regard to the morpho-functional plasticity of BAT and its dynamics during hypothyroidism. To that end, we used the antithyroid drug methimazole to induce hypothyroidism in rats for 7, 15, and 21 days. To reveal the remodeling of redox homeostasis, we analyzed protein expression of CuZn- and Mn-superoxide dismutase (CuZnSOD and MnSOD, respectively), catalase (CAT), glutathione peroxidase (GSH-Px), glutamyl–cysteine ligase (GCL), and thioredoxin (Trx); the activity of CuZnSOD, MnSOD, CAT, thioredoxin reductase (TR), glutathione reductase (GR), and GSH-Px; and total GSH content, as well as tissue protein localization patterns of AD enzymes. Furthermore, to examine changing redox homeostasis concerning structural remodeling associations, we investigated ultrastructural changes and protein localization at the subcellular level. Special attention was paid to the transcriptional regulation and participation of a master regulator of redox-metabolic reprogramming, Nrf2.

## 2. Materials and Methods

### 2.1. Animals and Experimental Design

All procedures performed in this experiment were approved by the Ethics Committee for the treatment of experimental animals of the Faculty of Biology at the University of Belgrade and by the Veterinary Directorate of the Ministry of Agriculture and Environmental Protection of the Republic of Serbia. Two-month-old Wistar rats (330 ± 30 g) were maintained under 12 h light/dark cycles at 22 ± 1 °C with ad libitum access to standard pelleted food. Animals were divided into four groups, each consisting of eight animals. To induce hypothyroidism, three groups were treated with the antithyroid drug methimazole (Methimazole crystalline M8506, Sigma-Aldrich Chemie GmbH, Munich, Germany) in drinking water (0.04%) for 7, 15, and 21 days, as previously described [46]. Animals in the fourth group drank tap water for 21 days and served as a euthyroid control.

### 2.2. Western Blotting

Following decapitation, interscapular BAT was isolated and prepared for Western blot as previously described [36]. Furthermore, protein concentration was determined by the method of Lowry et al. [47]. Primary antibodies against: CuZnSOD (0.2 µg mL^−1^; ab13498), MnSOD (0.2 µg mL^−1^, ab13533), CAT (1 µg mL^−1^; ab1877), GSH-Px (1 µg mL^−1^; ab17926), GCL (1 µg mL^−1^, ab17926), Trx (1 µg mL^−1^, ab26320), Nrf2 (1 µg mL^−1^, ab31163), and β-actin (0.5 µg mL^−1^; ab8226) were purchased from Abcam (Cambridge, UK). ImageJ software (National Institute of Health, Bethesda, MD, United States) was used for densitometric analyses of immunoreactive bands. Original band density was calculated as the sum of pixel intensities within a band using a gel analyze function. Final band density was obtained as the area under the peak per lane for the target protein averaged against β-actin as a gel loading control (band intensity for target protein)/(band intensity for β-actin). Protein expression is shown as mean +/− SEM and expressed as percentages against the control where the control group was taken as 100% ((mean for experimental group)/(mean for the control group) × 100).

### 2.3. The activity of Antioxidant Defense Enzymes

Total SOD activity was determined by a modified method of Misra and Fridovich [48]. For the determination of MnSOD activity, the assay was performed after the preincubation of protein samples with 4 mM KCN. CuZnSOD activity was calculated as the difference between total SOD and MnSOD activities. Enzyme activity was expressed in U mg^−1^ protein. CAT was assayed as suggested by the supplier (Sigma-Aldrich, St. Louis, MO, USA). The reaction was based on the H_2_O_2_ degradation rate by the action of CAT contained in the examined samples. The CAT activity was measured spectrophotometrically and expressed in mM H_2_O_2_ min^−1^ mg^−1^ protein. TR activity was assayed according to Luthman and Holmgren, and its specific activity was expressed in nmol NADPH min^−1^ mg^−1^ protein [49]. GR activity was assayed as suggested by Glatzle et al. and expressed in nmol NADPH min^−1^ mg^−1^ protein [50]. GSH-Px was determined with t-butylhydroperoxide as a substrate, and the activity was expressed in nmol NADPH min^−1^ mg^−1^ protein [51]. GSH content was examined in tissue samples after deproteinization with sulfosalicylic acid. Total GSH content was measured by enzyme-recycling assay after Griffith [52] and expressed in nmol GSH g^−1^ tissue.

### 2.4. Transmission Electron Microscopy & Immunogold Labeling

Immediately after dissection, small parts of interscapular BAT were fixed with 2% glutaraldehyde/2% paraformaldehyde in 0.1 M Sørensen phosphate buffer (PB, pH 7.2) for 1 h at 4 °C. After fixation, tissue was washed in phosphate buffer and then preincubated in 0.1% 3,3′-diaminobenzidine (DAB) in 0.1 M Sørensen phosphate buffer for 30 min, for peroxisomes staining. Furthermore, 0.01% H_2_O_2_ was added to the preincubation medium and incubated for 1 h at 37 °C. After rinsing in phosphate buffer, tissue was postfixed in 2% osmium tetraoxide in the same buffer, then routinely dehydrated using increasing concentrations of ethanol and embedded in Araldite. Ultra-thin sections of BAT were obtained using a Leica UC6 ultramicrotome (Leica Microsystems, Wetzlar, Germany) and mounted on nickel grids. After antigen retrieval in 10 mM citrate buffer and incubation with 5% bovine serum albumin (BSA) in Tris-buffered saline/0.1% Tween 20 (TBS-T) for 1 h at room temperature, grids were incubated overnight at 4 °C with primary antibodies against CuZnSOD (1:50, ab13498), MnSOD (1:100, ab13533), CAT (1:250, ab1877), GSH-Px (1:100, ab17926), Nrf2 (1:30, ab31163), and 4-hydroxynonenal (4-HNE, 1:1000, ab48506). After rinsing, grids were incubated with a 10-nm gold-conjugated anti-rabbit secondary antibody (1:20, ab27234) or a 10-nm gold-conjugated anti-mouse secondary antibody for 4-HNE (1:20, ab27241) for 1 h at room temperature. Sections were examined on a Philips CM12 transmission electron microscope (Philips/FEI, Eindhoven, The Netherlands) equipped with the digital camera SIS MegaView III (Olympus Soft Imaging Solutions, Münster, Germany).

### 2.5. Immunofluorescence

Semi-thin sections of interscapular BAT were used for standard immunolabeling procedure, using primary antibodies against Nrf2, 4-HNE, and appropriate fluorochrome-conjugated secondary antibody (1:400; Alexa Fluor^®^ 488 goat anti-rabbit for Nrf2, A11008, or Alexa Fluor^®^ 488 goat anti-mouse for 4-HNE, A11001; Thermo Fisher Scientific, Waltham, MA, USA). DAPI (1 μL mL^−1^, Sigma-Aldrich, Germany) was used for nuclei counterstaining. Slides were mounted with Mowiol (Polysciences, Eppelheim, Germany), and confocal images were acquired with a Leica TSC SP8 confocal microscope (Leica Microsystems) using 63/1.4 NA oil immersion lens. The specificity of immunofluorescence was tested by the omission of the primary antibody.

### 2.6. Light Microscopy and Immunohistochemistry

A piece of the interscapular BAT was fixed in 4% paraformaldehyde in phosphate buffer (pH 7.4) for 12 h. After overnight rinsing in tap water, samples were dehydrated through a series of increasing concentrations of ethanol and routinely embedded in paraffin. Series of 5 µm thick BAT sections were deparaffinized, rehydrated, and treated by heat-induced epitope retrieval—HIER procedure (citrate buffer pH = 6 in a microwave oven for 21 min, maximum power 800 W). Subsequently, sections were incubated with Nrf2 antibody (1:100, ab31163) overnight. Immunohistochemical reactions were developed with a commercial kit (Thermo Scientific Lab Vision Quanto HRP DAB TL-125-QHD) with DAB as a chromogen. Sections were counterstained with the Meyer hematoxylin (Biognost, HEMML-OT-1L), dehydrated, and mounted with DPX (mounting medium for histology, Sigma). All analyses were done with a Leica DMLB microscope equipped with a DFC295 camera (Leica Microsystems).

### 2.7. Statistics

All obtained data were analyzed by the GraphPad Prism software (GraphPad Prism, Version 5.03). Normal distribution was tested by D’Agustino and Pearson tests. If normality criteria were met, one-way ANOVA with post hoc Tukey’s multiple comparison test was run; if normality criteria were not met, Kruskal–Wallis non-parametric test was run. Statistical significance was accepted at *p* < 0.05.

## 3. Results

### 3.1. Hypothyroidism-Related Changes in Protein Expression of CuZnSOD, MnSOD, CAT, GSH-Px, GCL, and Trx

**Figure 1:**
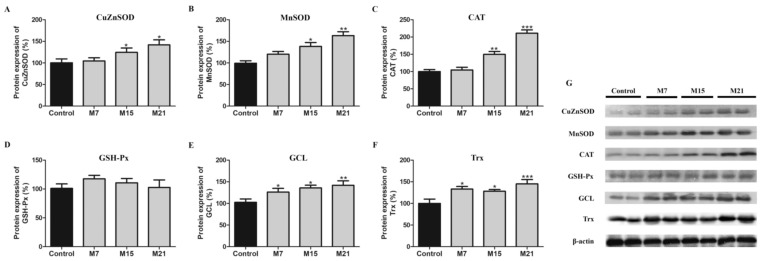
Protein expression of CuZnSOD (**A**), MnSOD (**B**), CAT (**C**), GSH-Px (**D**), GCL (**E**), Trx (**F**) in rat brown adipose tissue of the control (black) and hypothyroid groups (gray) treated with methimazole for 7 (M7), 15 (M15), and 21 (M21) days, respectively. The protein content is expressed as a percentage from the control. Band images from a representative blot of three trials are shown (**G**). Bars represent the mean ± SEM. Compared to control, * *p* < 0.05, ** *p* < 0.01, *** *p* < 0.001. Abbreviations: CuZn- and Mn-superoxide dismutase (CuZnSOD and MnSOD, respectively), catalase (CAT), glutathione peroxidase (GSH-Px), glutamyl–cysteine ligase (GCL), thioredoxin (Trx).

In general, hypothyroidism leads to increased protein expression of antioxidant defense enzymes in rat BAT (Figure 1). CuZnSOD, MnSOD, and CAT protein expression were increased on the 15th and 21st day, while GCL and Trx protein expression were increased from the 7th day to the end of the methimazole treatment, compared to control. GSH-Px protein expression was not affected by methimazole treatment.

### 3.2. Hypothyroidism-Related Changes in Enzyme Activity of CuZnSOD, MnSOD, CAT, TR, GR, GSH-Px, and Content of GSH

**Figure 2:**
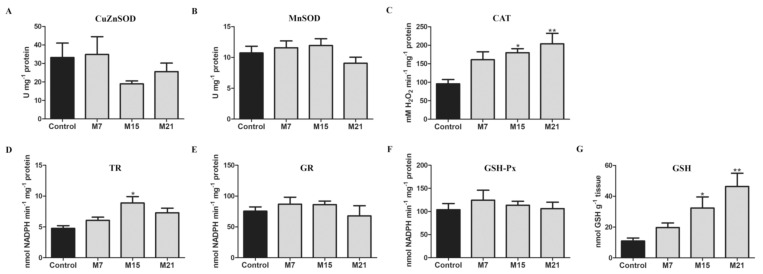
The activity of CuZnSOD (**A**), MnSOD (**B**), CAT (**C**), TR (**D**), GR (**E**), GSH-Px (**F**), and GSH content (**G**) in rat brown adipose tissue of the control (black) and hypothyroid groups (gray) treated with methimazole for 7 (M7), 15 (M15), and 21 (M21) days, respectively. Enzyme activity is expressed in absolute units in U mg-1 protein (A, B), mM H_2_O_2_ min^−1^ mg^−1^ protein (**C**), nM NADPH min^−1^ mg^−1^ protein (**D**–**F**), and nmol GSH g^−1^ tissue (**G**). Bars represent the mean ± SEM. Compared to control, * *p* < 0.05, ** *p* < 0.01. Abbreviations: CuZn- and Mn-superoxide dismutase (CuZnSOD and MnSOD, respectively), catalase (CAT), thioredoxin reductase (TR), glutathione reductase (GR), glutathione peroxidase (GSH-Px), glutathione (GSH).

Our results showed no significant differences in CuZnSOD, MnSOD, GSH-Px, and GR activities during methimazole treatment compared to the control (Figure 2). In contrast, CAT activity was distinctly increased on the 15th and 21st day. Moreover, total GSH content was increased on the 15th (threefold) and 21st day (fivefold) of methimazole treatment, compared to the control. The activity of TR was increased on the 15th day, compared to the control.

### 3.3. Changes in Subcellular Distribution of CuZnSOD, MnSOD, CAT, and GSH-Px during Hypothyroidism

**Figure 3:**
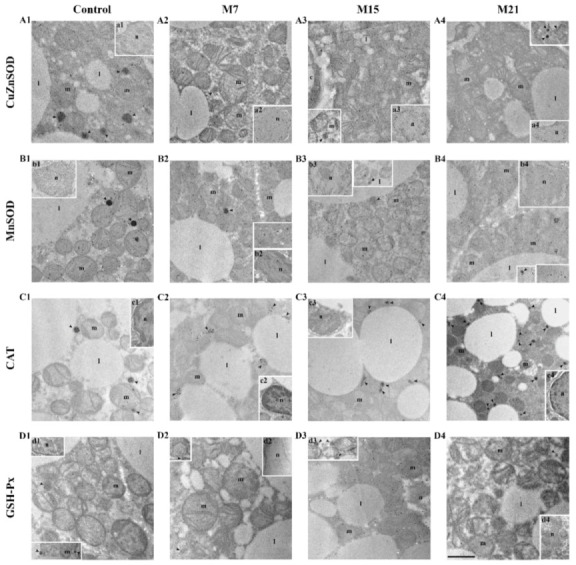
Immunogold labeling of CuZnSOD (**A1**–**A4**); MnSOD (**B1**–**B4**); CAT (**C1**–**C4**); and GSH-Px (**D1**–**D4**) in rat brown adipose tissue of the control and hypothyroid groups treated with methimazole for 7 (M7), 15 (M15), and 21 (M21) days, respectively. Insets, nuclear immunopositivity on CuZnSOD (a1-4), MnSOD (b1-4), CAT (c1-4), GSH-Px (d1, 2, 4); peroxisomal and mitochondrial immunopositivity (d3). l, lipid body; m, mitochondria; n, nuclei; arrowhead, peroxisomes. Scale bars 1 μm. Abbreviations: CuZn- and Mn-superoxide dismutase (CuZnSOD and MnSOD, respectively), catalase (CAT), glutathione peroxidase (GSH-Px).

The presence and specific organellar localization/compartmentalization of AD enzymes in brown adipocytes were detected by immunogold labeling. Namely, pre-existing CuZnSOD cytoplasmic localization characteristics for the euthyroid control persisted during methimazole treatment (Figure 3A1). However, hypothyroidism induced a newly established CuZnSOD localization in nuclei, at mitochondrial cristae, and at the peroxisomal membrane in brown adipocytes (Figure 3A2–A4). Interestingly, the strongest CuZnSOD immunolabeling was observed in the heterochromatin regions of the nuclei (Figure 3, insets a1–4), while cytoplasmic immunolabeling was observed in the form of clusters (Figure 3A1–A4).

Immunogold labeling of MnSOD confirmed its pre-existing mitochondrial localization in brown adipocytes of the euthyroid control (Figure 3B1). Hypothyroidism induced the appearance of highly MnSOD immunopositive mitochondria located in close proximity to and around lipid bodies on the 15th and 21st day (Figure 3B3,B4). Besides MnSOD localization at the mitochondrial outer membrane and cristae, hypothyroidism increased the presence of MnSOD in the mitochondrial matrix. Specific, circular clusterization of immunogold particles at the mitochondria was observed in both the control and hypothyroid groups. In addition to mitochondrial localization, MnSOD was also found in the nuclei of brown adipocytes in the control and hypothyroid groups (Figure 3, insets b1–4).

**Figure 4:**
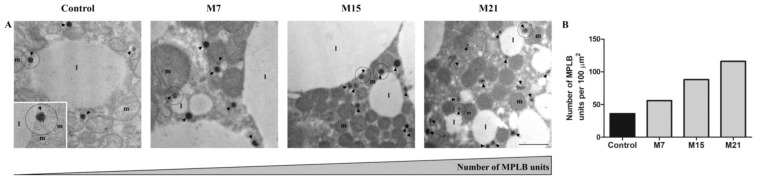
Close structural association of mitochondria, peroxisomes, and lipid bodies–MPLB units in brown adipocytes of control and hypothyroid groups treated with methimazole for 7 (M7), 15 (M15), and 21 (M21) days, respectively. At the electron micrographs, MPLB units were represented by circles (**A**), and their number increased with the time course (**B**). The number of MPLB units were presented as relative to 100 µm^2^ of cell area. Additionally, immunogold labeling of catalase (CAT) was shown. l, lipid body; m, mitochondria; n, nuclei; arrowhead, peroxisomes. Scale bar 1 μm.

CAT immunolabeling confirmed its pre-existing peroxisomal localization in the control and hypothyroid groups (Figure 3C1–C4). Immunopresence of CAT in peroxisomal matrix increased over the time course of methimazole treatment. Simultaneously, a growing number of newly established associations of peroxisomes, mitochondria, and lipid bodies were observed (Figure 4A, circles). This type of close structural association of mitochondria, peroxisomes, and lipid bodies (Figure 4A, inset) was also present in the control group, but hypothyroidism raised their number 1.55-, 2.44-, and 3.22-fold after 7, 15, and 21 days of methimazole treatment, respectively (Figure 4B). Along with that, hypothyroidism induced more intense cytoplasmic localization of CAT starting from the 15th day of methimazole treatment (Figure 3C3,C4). Weak CAT immunopresence was also found in the mitochondria and nuclei in both the control and hypothyroid groups (Figure 3C1–C4, and insets c1–4), with no differences in the level or localization in their respective subcompartments (nuclear hetero- vs. euchromatin or mitochondrial membrane vs. matrix).

GSH-Px immunolabeling showed its pre-existing localization in all three mitochondrial subcompartments (mitochondrial outer membrane, cristae, and matrix), cytoplasm, and peroxisomes in the control group, which remained the same during the time course of the treatment (Figure 3D1–D4). The newly established localization of GSH-Px induced by hypothyroidism was found at lipid bodies and around them (at the level of lipid body monolayered membrane) on the 15th day, and remained at the same level until the end of the treatment (Figure 3D3,D4). We observed a nuclear presence of GSH-Px without differences in the level or localization in nuclear subcompartments (hetero- vs. euchromatin) (Figure 3D3 and insets d1, d2, d4).

### 3.4. Protein Expression of Nrf2 in BAT during Hypothyroidism

**Figure 5:**
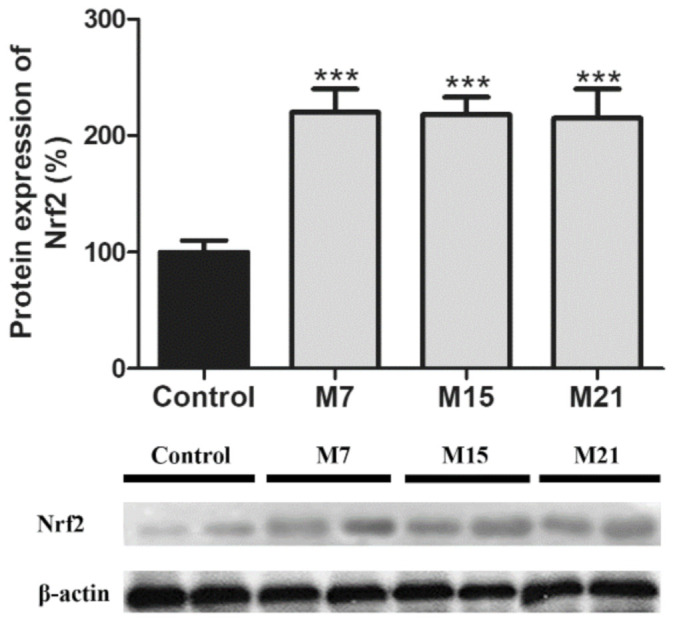
Protein expression of nuclear factor erythroid 2-related factor 2 (Nrf2) in rat brown adipose tissue of the control (black) and hypothyroid groups (gray) treated with methimazole for 7 (M7), 15 (M15), and 21 (M21) days, respectively. The protein content is expressed as a percentage from the control. Band images from a representative blot of three trials are shown. Bars represent the mean ± SEM. Compared to control, *** *p* < 0.001.

Nrf2 protein content (Figure 5) increased on the 7th day of treatment and was maintained at the same level for 15 and 21 days of treatment, compared to the control.

**Figure 6:**
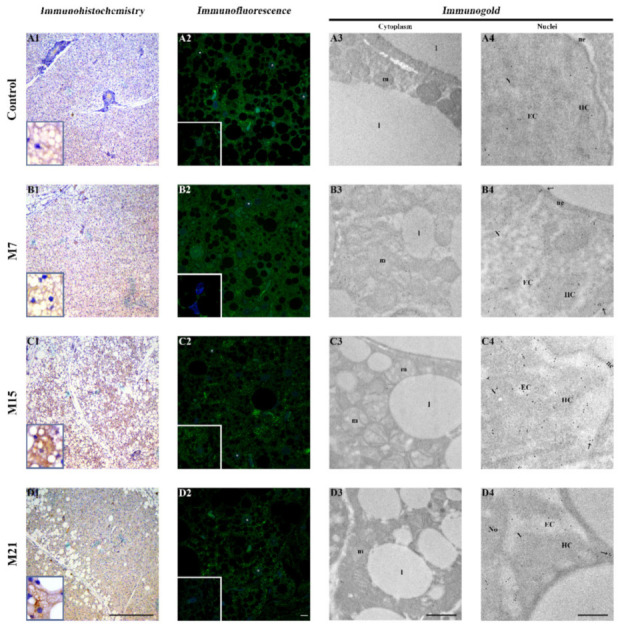
Nuclear factor erythroid 2-related factor 2 (Nrf2) immunoexpression in rat brown adipocytes. Immunohistochemistry, immunofluorescent, and immunogold labeling of Nrf2 in rat brown adipose tissue of the control (**A1**–**A4**) and hypothyroid groups M7 (**B1**–**B4**), M15 (**C1**–**C4**), and M21 (**D1**–**D4**), respectively. l, lipid body; m, mitochondria; N, nucleus; No, nucleolus; EC, euchromatin; HC, heterochromatin; ne, nuclear envelope; white star, immunopositive nuclei (confocal microscopy); arrows, nuclear pore complex (TEM). Scale bars: light microscopy 200 μm, confocal microscopy 5 μm on x2.5 zoom, transmission electron microscopy 1 μm.

In parallel, an increasing Nrf2 immunopositivity was observed from 7 to 21 days of treatment in the cytoplasm and some nuclei (insets) of brown adipocytes (Figure 6A1–D1). Immunofluorescent labeling confirmed Nrf2 presence in both cell compartments, but also revealed immunopositivity in subcompartments: mitochondria and peroxisomes in cytoplasm and chromatin in the nucleus (Figure 6A2–D2). Additionally, strong perinuclear immunopositivity was also observed. Immunogold labeling further corroborated these findings; Nrf2 was found in brown adipocytes’ nuclei (Figure 6B4–D4), located predominantly in heterochromatin regions, at the level of the nuclear envelope and within the nuclear pore complex (Figure 6B4–D4, arrows). Furthermore, the immunopositive reaction was localized at the mitochondrial outer membrane and mitochondria cristae (Figure 6A3–D3).

### 3.5. Tissue and Cell Distribution Patterns of 4-HNE-Modified Proteins during Hypothyroidism

**Figure 7:**
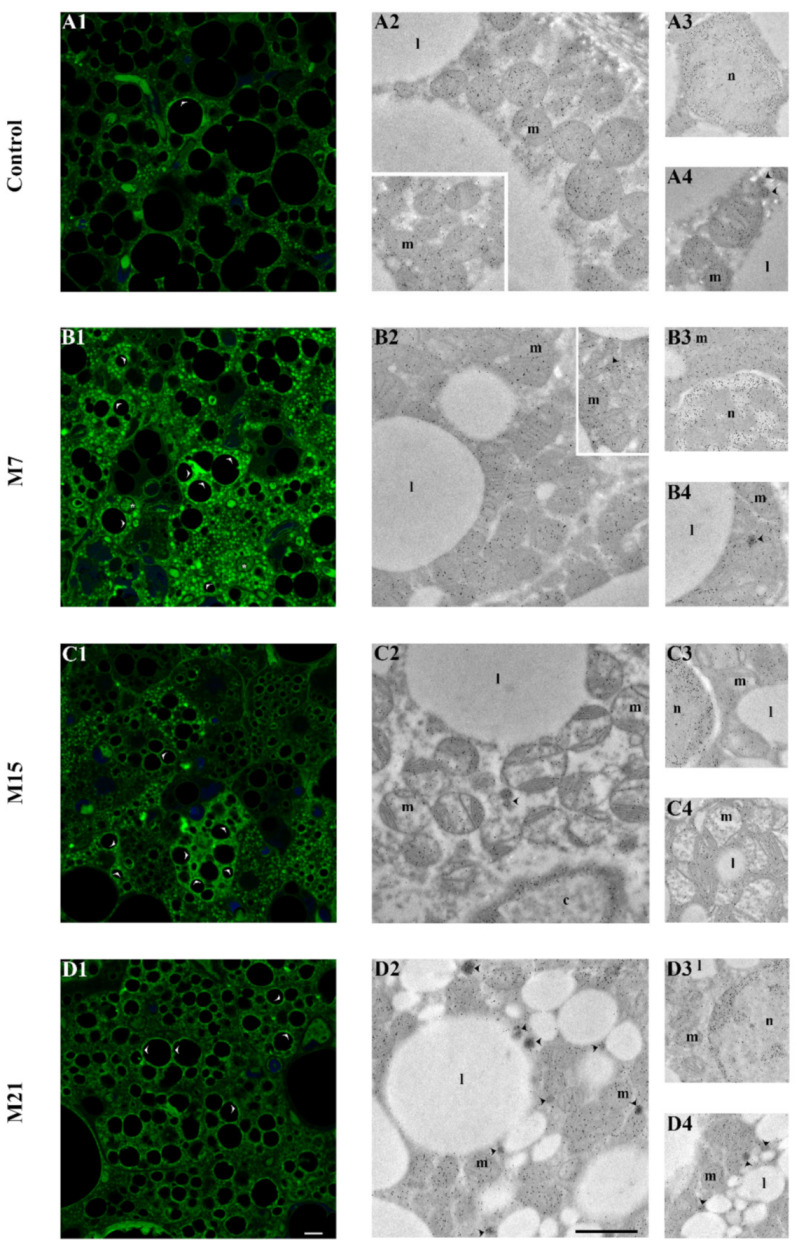
Immunofluorescent and immunogold labeling of 4-hydroxynonenal (4-HNE) modified proteins in rat brown adipose tissue of control (**A1**–**A4**) and hypothyroid groups M7 (**B1**–**B4**), M15 (**C1**–**C4**), and M21 (**D1**–**D4**), respectively. l, lipid body; m, mitochondria; n, nuclei; c, blood capillary; black arrowhead, peroxisomes; white arrowhead, immunopositive reaction around lipid bodies (confocal microscopy); (*) white star, immunopositive nuclei (confocal microscopy). Scale bars: confocal microscopy 5 μm on x2.5 zoom, transmission electron microscopy 1 μm.

As compared to the control, higher immunopositivity of 4-HNE was observed on the 7th day of methimazole treatment (Figure 7A–D1). The strongest immunopositive reaction was localized in the mitochondria and around lipid bodies, while a less prominent reaction was observed in the cytoplasm and some nuclei (Figure 7B1). Closer examination of 4-HNE-modified proteins by immunogold labeling (Figure 7) showed an immunopositive reaction localized at the mitochondrial cristae, peroxisomal membrane, and around lipid bodies (at their monolayered membrane), as well as in the nuclear euchromatin region (Figure 7A2–A4) in the control and hypothyroid groups. However, in BAT of 7 days treated rats, higher 4-HNE reaction was observed in the peroxisomal matrix (Figure 7B2–B4). From the 15th day until the end of the treatment, a strong immunopositive reaction was also observed at the outer membrane of the mitochondria and the heterochromatin region of nuclei (Figure 7C2–C4,D2–D4).

## 4. Discussion

This study revealed that hypothyroidism changes the protein expression patterns, activities, and cellular localization of AD enzymes alongside the unique structural reorganization in brown adipocytes. Analysis of BAT showed a time-dependent increase in protein expression and content of investigated enzymatic and non-enzymatic AD components during hypothyroidism. Immunogold labeling revealed specific AD enzyme localization patterns at the cellular level. Namely, in addition to their pre-existing localization, AD enzymes were found in newly established cellular localizations—CuZnSOD, MnSOD, CAT, and GSH-Px in the nucleus and CuZnSOD and MnSOD in peroxisomes, while GSH-Px was found in and around lipid bodies (membrane monolayer). The widening localization of AD enzymes was accompanied by the reorganization of cell compartments and the establishment of specific structural associations of mitochondria, peroxisomes, and lipid bodies (MPLB units). Furthermore, we showed that the Nrf2 signaling pathway might play a role in the orchestration and control of BAT redox remodeling, while its mitochondrial localization suggests additional roles in metabolic and structural remodeling. The results indicate that spatiotemporal overlap in the remodeling of AD is orchestrated by Nrf2, and suggest the potential mechanism of redox reprogramming in BAT during hypothyroidism.

### 4.1. Hypothyroidism Induces Antioxidant Defense in BAT

Previous studies of AD in different tissues and different models of hypothyroidism yielded contradictory results. Most studies showed a general trend towards the upregulation of antioxidant defense, evident as an increase in AD enzyme activity in BAT [46], brain [53], and erythrocytes of hypothyroid rats [54], as well as in the serum and erythrocytes of patients with hypothyroidism [55,56,57]. However, some authors reported unchanged AD enzyme activity in the serum and erythrocytes of hypothyroidism patients [56,58,59], while others have reported decreased AD enzyme activities in isolated macrophages [60], lymphoid organs and muscles [61], liver [62], and in the serum of hypothyroid rats [63]. Additionally, some previous studies showed that hypothyroidism activates BAT function [28,44] similarly to cold [35,37,64,65]. Cold-induced thermogenic activation of BAT is concordant with the upregulation of AD [37]. In a similar way, our results show that hypothyroidism in BAT leads to widespread changes in AD level. Namely, we showed that hypothyroidism increased protein expression of AD enzymes (CuZnSOD, MnSOD, CAT, GCL, Trx, and total GSH content) and TR and CAT activity in BAT. A marked increase in CAT protein expression and activity could reflect the metabolic activation of mitochondrial and peroxisomal oxidative metabolism, indicative of BAT activation. This is further supported by a simultaneous increase in GSH content and protein expression of GCL, the rate-limiting enzyme in GSH synthesis. Thus, as in the adaptation of BAT to cold [35], the peroxidative and GSH-dependent parts of AD could be particularly indicative of the activation of BAT in hypothyroidism. We did not observe changes in CuZnSOD and MnSOD activity alongside their increased protein expression, which could be a result of various posttranslational modifications, including allosteric regulation and reversible covalent modification such as oxidation, phosphorylation, acetylation, methylation, etc.

### 4.2. Hypothyroidism Induces the Creation of Specific Organellar Units

Large-scale changes in redox-metabolic homeostasis are often followed by a functional reorganization of different cell compartments that includes structural remodeling in response to changing tissue demands. In this context, structural remodeling implies the creation of close structural associations of organelles whose primary purpose is to support specific functional adaptations. This study revealed that hypothyroidism induces a close structural association of mitochondria, peroxisomes, and lipid bodies—MPLB units. Functional interplay between peroxisomes and mitochondria during the β-oxidation of fatty acids and ROS removal is a striking example of an interorganellar codependent relationship [66,67,68]. It was also shown that peroxisome-derived lipids regulate thermogenesis by mediating cold-induced mitochondrial dynamic [69]. Here, we showed that peroxisomes are the most numerous around lipid bodies, which is a morphological indication of their role in lipid metabolism. Previous works also found that peroxisomes associate with lipid bodies in 3T3-L1 adipocytes, mouse epididymal WAT [70], yeast [71], and oil-rich plant seeds [72]. Bidirectional lipid trafficking between peroxisomes and lipid bodies controls lipid metabolic flux and the balance between energy production and utilization depending on the metabolic context [73], while a close interaction between these two organelles suggests a coordinated regulation of metabolism and lipid trafficking across their membranes [74]. However, intracellular regulation of lipid trafficking involves mitochondria, besides peroxisomes and lipid bodies, where under various metabolic conditions, contacts between lipid bodies and mitochondria serve as sites for both lipogenesis and lipolysis [75]. Our results are in line with previous research showing that lipid-body-associated mitochondria constitute a specific population of mitochondria, which, together with peroxisomes, form MPLB structural/functional units in brown adipocytes. We hypothesize that the newly emerged structural unity of the major functional actors, followed by a specific AD enzyme localization pattern, suggests a unique functional syncytium that supports the redox-dependent changes induced by hypothyroidism. Thus, hypothyroidism induces concomitant structural remodeling of BAT with redox homeostasis changes. Hence, our results indicate strong and tightly regulated cooperation between different cell compartments due to the tissue’s adaptive response to a newly established homeostatic state on the 15th and 21st day of hypothyroidism.

### 4.3. Hypothyroidism-Induced Newly Established Localization of AD Enzymes

Considering the aforementioned changes of AD enzyme expression patterns, we further analyzed their distribution pattern to reveal possible hypothyroidism-induced changes. Using immunogold labeling, we found their presence in pre-existing but also in new locations at the subcellular level. One of these new locations was brown adipocyte nuclei, with a pronounced accumulation of analyzed AD enzymes over the time course of hypothyroidism. The question of their function in this compartment remains open, whether it is the same as in pre-existing localizations (antioxidant and protective role), or they perform a different function in nuclei, like moonlighting proteins (regulatory role). The observed differences in AD enzymes residing subcompartments (hetero- and euchromatin), temporal occurrence (7, 15 and/or 21 days), and correlation with their expression and activity favor additional antioxidant protective roles. However, it would be interesting to examine whether AD enzymes perform novel functions in the nucleus of brown adipocytes.

It is widely accepted that protein localization is strongly related to functional compartmentalization within a cell, and therefore particular protein localization could indicate an increased need for their activity at that specific cell compartment. For example, the localization of AD enzymes in peroxisomes, MnSOD in particular, and the continued increase in peroxisomal CAT protein expression during hypothyroidism could indicate a synergistic response of redox remodeling and the remodeling of oxidative processes [74,76]. That could be the main reason for the structural association of mitochondria, peroxisomes, and lipid bodies (MPLB), and why they organize as a functional unit. In addition, we found specific AD enzyme redistribution patterns: CuZnSOD in mitochondria at the mitochondrial cristae and peroxisomal membrane; MnSOD in the peroxisomal matrix, CAT in the mitochondria, and GSH-Px in and around lipid bodies (membrane monolayer), suggesting enzyme subcompartmentalization in fine-tuning of AD in brown adipocytes during hypothyroidism.

### 4.4. Transcriptional Control of Brown Adipose Tissue Redox Remodeling Involves Nrf2

Transcription factor Nrf2 is a master regulator of redox homeostasis regulating the expression of genes involved in phase II detoxification and antioxidant defense. Among those genes are genes encoding for antioxidant enzymes and proteins, including CuZnSOD, MnSOD, CAT, GSH-Px, GCL, and Trx [77,78,79,80,81]. Moreover, Nrf2 integrates redox and metabolic homeostasis by regulating the expression of hundreds of genes involved in the intermediate metabolism, transcription factors, mitochondrial function, cell cycle, and other cellular cytoprotective pathways [15,16,17,18]. Our results show that hypothyroidism induces Nrf2 protein expression and nuclear translocation in BAT. Subsequently, Nrf2 in BAT probably triggers a signaling cascade that positively regulates AD gene expression, as evident from the increased CuZnSOD, MnSOD, CAT, GCL, and Trx protein expression.

Regarding Nrf2 subcellular localization, the presence of Nrf2 in the nuclei of brown adipocytes in euthyroid control indicates its regulatory role under physiological conditions. During methimazole treatment, an increase in Nrf2 nuclear immunolocalization, especially on the 15th day, indicates that Nrf2 modulates redox homeostasis in BAT. Moreover, activation of the Nrf2 pathway in BAT could provide the molecular basis not only for comprehensive changes in the antioxidant defense but for the changes at the ultrastructural level. Recent studies highlight that Nrf2 is directly involved in the regulation of mitochondrial function and dynamics [82,83,84,85,86,87,88,89]. The first physical association of Nrf2 and the mitochondrial outer membrane was reported in 2008 [90], and it was proposed that the direct interaction of Nrf2 with mitochondria serves the purpose of mitochondrial integrity and function preservation [91,92]. Here, we show Nrf2 presence at both the outer and inner mitochondrial membrane. Whether the localization of Nrf2 at the mitochondrial inner membrane indicates another new role for this transcription factor remains to be investigated.

### 4.5. Possible Mechanism of Redox-Structural Remodeling in Brown Adipose Tissue during Hypothyroidism through Secondary Messengers of Redox Signaling

This study showed increased Nrf2 protein expression after 7 days of hypothyroidism, followed by upregulation of AD enzyme protein expression on the 7th, 15th, and 21st day of hypothyroidism. Interestingly, the strongest immunopresence of 4-HNE was also observed on the 7th day. 4-HNE, as an end-product of lipid peroxidation, regulates both cell survival or death, depending on its concentration, cell type, and cellular metabolic circumstances [93]. Recent in vitro and in vivo studies demonstrated that 4-HNE induces the nuclear translocation of Nrf2, acting as a secondary messenger of redox signaling [94,95,96,97]. Furthermore, 4-HNE appears to cross membranes readily, and therefore, 4-HNE protein adducts can be localized in all cell compartments [98]. Accordingly, increased immunopresence of 4-HNE and specific localizations in nuclei, mitochondria, peroxisomes, cytoplasm, and around lipid bodies suggests multiple roles of 4-HNE, including Nrf2 activation in BAT during hypothyroidism. The transient increase in immunopositivity for 4-HNE at the beginning of the treatment suggests its potential role as a redox signaling messenger. An increase in 4-HNE indicates changes in redox homeostasis apropos of increased prooxidative pressure, leading to the initiation of the antioxidant response, reflected in the increased protein expression of Nrf2 and AD enzymes. This antioxidant cascade leads to the establishment of a new homeostatic state, accompanied by the recurrence of 4-HNE to the control level.

## 5. Conclusions

In conclusion, this study revealed comprehensive redox and structural remodeling of BAT in hypothyroidism. Induction of AD, accompanied by subcellular relocalization of AD enzymes, is tied to structural reorganization and organellar remodeling. Such organellar remodeling establishes a close structural association between mitochondria, peroxisomes, and lipid bodies—MPLB units, and reflects the need for functional cooperation to maintain redox homeostasis. Moreover, an increase in Nrf2 protein expression indicates a potential role of this redox-sensitive transcription factor in the redox-structural remodeling of BAT, with the possible involvement of a secondary messenger of redox signaling, 4-HNE. How these changes are related to the thermogenic and metabolic activity of BAT in hypothyroidism will be in the focus of our future research.

## Data Availability

Not applicable

## References

[1] Chiovato L., Magri F., Carlé A. (2019). Hypothyroidism in Context: Where We’ve Been and Where We’re Going. Adv. Ther..

[2] Brenta G. (2011). Why Can Insulin Resistance Be a Natural Consequence of Thyroid Dysfunction?. J. Thyroid Res..

[3] Chung G.E., Kim D., Kim W., Yim J.Y., Park M.J., Kim Y.J., Yoon J.H., Lee H.S. (2012). Non-alcoholic fatty liver disease across the spectrum of hypothyroidism. J. Hepatol..

[4] Sinha R.A., Singh B.K., Yen P.M. (2014). Thyroid hormone regulation of hepatic lipid and carbohydrate metabolism. Trends Endocrinol. Metab..

[5] Martinez B., Ortiz R.M. (2017). Thyroid hormone regulation and insulin resistance: Insights from animals naturally adapted to fasting. Physiology.

[6] Halliwell B. (2007). Biochemistry of oxidative stress. Biochem. Soc. Trans..

[7] Roberts C.K., Sindhu K.K. (2009). Oxidative stress and metabolic syndrome. Life Sci..

[8] Xing M. (2012). Oxidative stress: A new risk factor for thyroid cancer. Endocr. Relat. Cancer.

[9] Bullon P., Newman H.N., Battino M. (2014). Obesity, diabetes mellitus, atherosclerosis and chronic periodontitis: A shared pathology via oxidative stress and mitochondrial dysfunction?. Periodontol. 2000.

[10] Palipoch S., Koomhin P. (2015). Oxidative Stress-Associated Pathology: A Review. Sains Malays..

[11] Mancini A., Di Segni C., Raimondo S., Olivieri G., Silvestrini A., Meucci E., Currò D. (2016). Thyroid Hormones, Oxidative Stress, and Inflammation. Mediat. Inflamm..

[12] Rani V., Deep G., Singh R.K., Palle K., Yadav U.C.S. (2016). Oxidative stress and metabolic disorders: Pathogenesis and therapeutic strategies. Life Sci..

[13] Halliwell B. (1996). Cellular Stress and Protection Mechanisms. Biochem. Soc. Trans..

[14] Sun Y., Oberley L.W. (1996). Redox regulation of transcriptional activators. Free Radic. Biol. Med..

[15] Lee J.M., Calkins M.J., Chan K., Kan Y.W., Johnson J.A. (2003). Identification of the NF-E2-related factor-2-dependent genes conferring protection against oxidative stress in primary cortical astrocytes using oligonucleotide microarray analysis. J. Biol. Chem..

[16] Suh J.H., Shenvi S.V., Dixon B.M., Liu H., Jaiswal A.K., Liu R.M., Hagen T.M. (2004). Decline in transcriptional activity of Nrf2 causes age-related loss of glutathione synthesis, which is reversible with lipoic acid. Proc. Natl. Acad. Sci. USA.

[17] Ryoo I., Kwak M.K. (2018). Regulatory crosstalk between the oxidative stress-related transcription factor Nfe2l2/Nrf2 and mitochondria. Toxicol. Appl. Pharmacol..

[18] Schmidlin C.J., Dodson M.B., Madhavan L., Zhang D.D. (2019). Redox regulation by NRF2 in aging and disease. Free Radic. Biol. Med..

[19] Dalle-Donne I., Scaloni A., Giustarini D., Cavarra E., Tell G., Lungarella G., Colombo R., Rossi R., Milzani A. (2003). Proteins as biomarkers of oxidattiv/e/nitrosative stress in diseases: The contribution of redox proteomics. Mass Spectrom. Rev..

[20] Margaritelis N.V., Cobley J.N., Paschalis V., Veskoukis A.S., Theodorou A.A., Kyparos A., Nikolaidis M.G. (2016). Going retro: Oxidative stress biomarkers in modern redox biology. Free Radic. Biol. Med..

[21] Korac B., Kalezic A., Pekovic-Vaughan V., Korac A. (2021). Redox changes in obesity, metabolic syndrome, and diabetes. Redox Biol..

[22] Cannon B., Nedergaard J. (2004). Brown adipose tissue: Function and physiological significance. Physiol. Rev..

[23] Ghorbani M., Himms-Hagen J. (1997). Appearance of brown adipocytes in white adipose tissue during CL 316,243-induced reversal of obesity and diabetes in Zucker fa/fa rats. Int. J. Obes..

[24] Rothwell N.J., Stock M.J. (1997). A role for brown adipose tissue in diet-induced thermogenesis. Obes. Res..

[25] Nedergaard J., Bengtsson T., Cannon B. (2007). Unexpected evidence for active brown adipose tissue in adult humans. Am. J. Physiol. Metab..

[26] Carey A.L., Kingwell B.A. (2013). Brown adipose tissue in humans: Therapeutic potential to combat obesity. Pharmacol. Ther..

[27] Saito M. (2014). Human brown adipose tissue: Regulation and anti-obesity potential. Endocr. J..

[28] Mory G., Ricquier D., Pesquiés P., Hémon P. (1981). Effects of hypothyroidism on the brown adipose tissue of adult rats: Comparison with the effects of adaptation to cold. J. Endocrinol..

[29] Rubio A., Raasmaja A., Silva J.E. (1995). Thyroid hormone and norepinephrine signaling in brown adipose tissue. II: Differential effects of thyroid hormone on b3-adrenergic receptors in brown and white adipose tissue. Endocrinology.

[30] Silva J.E. (1995). Thyroid Hormone Control of Thermogenesis and Energy Balance. Thyroid.

[31] Bianco A.C., McAninch E.A. (2013). The role of thyroid hormone and brown adipose tissue in energy homoeostasis. Lancet Diabetes Endocrinol..

[32] Hsieh A.C.L., Carlson L.D. (1956). Role of the Thyroid in Metabolic Low Temperature. Am. J. Physiol. Leg. Content.

[33] Abelenda M., Puerta M.L. (1990). Cold-induced thermogenesis in hypothyroid rats. Pflügers Arch. Eur. J. Physiol..

[34] Zaninovich A.A., Raíces M., Rebagliati I., Ricci C., Hagmüller K. (2002). Brown fat thermogenesis in cold-acclimated rats is not abolished by the suppression of thyroid function. Am. J. Physiol. Endocrinol. Metab..

[35] Laurberg P., Andersen S., Karmisholt J. (2005). Cold Adaptation and Thyroid Hormone Metabolism. Horm.

[36] Petrović V., Korać A., Buzadžić B., Korać B. (2005). The effects of L-arginine and L-NAME supplementation on redox-regulation and thermogenesis in interscapular brown adipose tissue. J. Exp. Biol..

[37] Petrović V., Buzadžić B., Korać A., Vasilijević A., Janković A., Korać B. (2006). Free radical equilibrium in interscapular brown adipose tissue: Relationship between metabolic profile and antioxidative defense. Comp. Biochem. Physiol. C Toxicol. Pharmacol..

[38] Petrović V., Buzadžić B., Korać A., Korać B. (2010). Antioxidative defense and mitochondrial thermogenic response in brown adipose tissue. Genes Nutr..

[39] Lettieri-Barbato D. (2019). Redox control of non-shivering thermogenesis. Mol. Metab..

[40] Suter E.R. (1969). The fine structure of brown adipose tissue. I. Cold-induced changes in the rat. J. Ultrasructure Res..

[41] Korac A., Buzadzic B., Petrovic V., Vasilijevic A., Jankovic A., Micunovic K., Korac B. (2008). The role of nitric oxide in remodeling of capillary network in rat interscapular brown adipose tissue after long-term cold acclimation. Histol. Histopathol..

[42] Petrović V., Korać A., Buzadžić B., Vasilijević A., Janković A., Mićunović K., Korać B. (2008). Nitric oxide regulates mitochondrial re-modelling in interscapular brown adipose tissue: Ultrastructural and morphometric-stereologic studies. J. Microsc..

[43] Petrović V., Buzadžić B., Korać A., Vasilijević A., Janković A., Korać B. (2010). NO modulates the molecular basis of rat interscapular brown adipose tissue thermogenesis. Comp. Biochem. Physiol. C Toxicol. Pharmacol..

[44] Dicker A., Raasmaja A., Cannon B., Nedergaard J. (1992). Increased α1-adrenoceptor density in brown adipose tissue indicates recruitment drive in hypothyroid rats. Am. J. Physiol. Endocrinol. Metab..

[45] Lapa C., Maya Y., Wagner M., Arias-Loza P., Werner R.A., Herrmann K., Higuchi T. (2015). Activation of brown adipose tissue in hypothyroidism. Ann. Med..

[46] Petrović N., Cvijić G., Davidović V. (2001). The activity of antioxidant enzymes and the content of uncoupling protein-1 in the brown adipose tissue of hypothyroid rats: Comparison with effects of iopanoic acid. Physiol. Res..

[47] Lowry O.H., Rosebrough N.J., Farr A.L., Randall R.J. (1951). Protein measurement with the folin phenol reagent. Anal. Biochem..

[48] Misra H.P., Fridovich I. (1972). The role of superoxide anion in the autoxidation of epinephrine and a simple assay for superoxide dismutase. J. Biol. Chem..

[49] Luthman M., Holmgren A. (1982). Rat Liver Thioredoxin and Thioredoxin Reductase: Purification and Characterization. Biochemistry.

[50] Glatzle D., Vuilleumier J.P., Weber F., Decker K. (1974). Glutathione reductase test with whole blood, a convenient procedure for the assessment of the riboflavin status in humans. Experientia.

[51] Paglia D.E., Valentine W.N. (1967). Studies on the quantitative and qualitative characterization of erythrocyte glutathione peroxidase. J. Lab. Clin. Med..

[52] Griffith O.W. (1980). Determination of Glutathione and Glutathione Disulfide Using Glutathione Reductase and 2-vinylpyridine. Anal. Biochem..

[53] Mano T., Sinohara R., Sawai Y., Oda N., Nishida Y., Mokuno T., Asano K., Ito Y., Kotake M., Hamada M. (1995). Changes in lipid peroxidation and free radical scavengers in the brain of hyper- and hypothyroid aged rats. J. Endocrinol..

[54] Yilmaz S., Ozan S., Benzer F., Canatan H. (2003). Oxidative damage and antioxidant enzyme activities in experimental hypothyroidism. Cell Biochem. Funct..

[55] Santi A., Duarte M.M.M.F., Moresco R.N., Menezes C., Bagatini M.D., Schetinger M.R.C., Loro V.L. (2010). Association between thyroid hormones, lipids and oxidative stress biomarkers in overt hypothyroidism. Clin. Chem. Lab. Med..

[56] Santi A., Duarte M.M.M.F., De Menezes C.C., Loro V.L. (2012). Association of lipids with oxidative stress biomarkers in subclinical hypothyroidism. Int. J. Endocrinol..

[57] Reddy V.S., Gouroju S., Suchitra M.M., Suresh V., Sachan A., Srinivasa Rao P.V.L.N., Bitla A.R. (2013). Antioxidant defense in overt and subclinical hypothyroidism. Horm. Metab. Res..

[58] Baskol G., Atmaca H., Tanriverdi F., Baskol M., Kocer D., Bayram F. (2007). Oxidative stress and enzymatic antioxidant status in patients with hypothyroidism before and after treatment. Exp. Clin. Endocrinol. Diabetes.

[59] Dave B.N., Paradkar N.M. (2009). Total superoxide dismutase, Cu/Zn superoxide dismutase and glutathione peroxidase in untreated hyperthyroidism and hypothyroidism. JK Sci..

[60] Pereira B., Fernando L., Costa R.B., Safi D.A., Bechara E.J., Curi R. (1995). Hormonal regulation of superoxide dismutase, catalase, and glutathione peroxidase activities in rat macrophages. Biochem. Pharmacol..

[61] Pereira B., Rosa L.F., Safi D.A., Bechara E.J., Curi R. (1994). Control of superoxide dismutase, catalase and glutathione peroxidase activities in rat lymphoid organs by thyroid hormones. J. Endocrinol..

[62] Cano-Europa E., Blas-Valdivia V., Lopez-Galindo G.E., Franco-Colin M., Pineda-Reynoso M., Hernandez-Garcia A., Ortiz-Butron R. (2010). Methimazole-Induced Hypothyroidism Causes Alterationof The REDOX Environment, Oxidative Stress, Andhepatic Damage; Events Not Caused By Hypothyroidism Itself. Ann. Hepatol..

[63] De Souza Cardoso J., Baldissarelli J., Reichert K.P., Teixeira F.C., Pereira Soares M.S., Chitolina Schetinger M.R., Morsch V.M., Farias Martins Filho A.O., Duarte Junior H.R., Ribeiro Coriolano F.H. (2021). Neuroprotection elicited by resveratrol in a rat model of hypothyroidism: Possible involvement of cholinergic signaling and redox status. Mol. Cell. Endocrinol..

[64] Nedergaard J., Alexson S., Cannon B. (1980). Cold adaptation in the rat: Increased brown fat peroxisomal β-oxidation relative to maximal mitochondrial oxidative capacity. Am. J. Physiol. Cell Physiol..

[65] Barja de Quiroga G., Lopez-Torres M., Perez-Campo R., Abelenda M., Paz Nava M., Puerta M.L. (1991). Effect of cold acclimation on GSH, antioxidant enzymes and lipid peroxidation in brown adipose tissue. Biochem. J..

[66] Fransen M., Nordgren M., Wang B., Apanasets O. (2012). Role of peroxisomes in ROS/RNS-metabolism: Implications for human disease. Biochim. Biophys. Acta Mol. Basis Dis..

[67] Lismont C., Nordgren M., Van Veldhoven P.P., Fransen M. (2015). Redox interplay between mitochondria and peroxisomes. Front. Cell Dev. Biol..

[68] Wanders R.J.A. (2014). Metabolic functions of peroxisomes in health and disease. Biochimie.

[69] Park H., He A., Min T., Johnson M.J., Dean M.J., Pietka T.A., Chen Y., Zhang X., Hsu F.-F., Razani B. (2018). Peroxisome-derived lipids regulate adipose thermogenesis by mediating cold-induced mitochondrial fission. J. Clin. Investig..

[70] Schrader M. (2001). Tubulo—Reticular Clusters of Peroxisomes in Living COS-7 Cells: Dynamic Behavior and Association with Lipid Droplets. J. Histochem. Cytochem..

[71] Binns D., Januszewski T., Chen Y., Hill J., Markin V.S., Zhao Y., Gilpin C., Chapman K.D., Anderson R.G.W., Goodman J.M. (2006). An intimate collaboration between peroxisomes and lipid bodies. J. Cell Biol..

[72] Kunze M., Pracharoenwattana I., Smith S.M., Hartig A. (2006). A central role for the peroxisomal membrane in glyoxylate cycle function. Biochim. Biophys. Acta.

[73] Bartz R., Li W., Venables B., Zehmer J.K., Roth M.R., Welti R., Anderson R.G.W., Liu P., Chapman K.D. (2007). Lipidomics reveals that adiposomes store ether lipids and mediate phospholipid traffic 1. J. Lipid Res..

[74] Lodhi I.J., Semenkovich C.F. (2014). Peroxisomes: A nexus for lipid metabolism and cellular signaling. Cell Metab..

[75] Olzmann J.A., Carvalho P. (2018). Dynamics and functions of lipid droplets. Nat. Rev. Mol. Cell Biol..

[76] Giacobino J.P., Moinat M., Muzzin P., Siegrist-Kaiser C.A., Seydoux J., Girardier L. (1989). Peroxisomal oxidative capacity of brown adipose tissue depends on the thyroid status. Mol. Cell. Endocrinol..

[77] Girnun G.D., Domann F.E., Moore S.A., Robbins M.E.C. (2002). Identification of a functional peroxisome proliferator-activated receptor response element in the rat catalase promoter. Mol. Endocrinol..

[78] Milani P., Gagliardi S., Cova E., Cereda C. (2011). SOD1 transcriptional and posttranscriptional regulation and its potential implications in ALS. Neurol. Res. Int..

[79] Kim Y.S., Vallur P.G., Phaëton R., Mythreye K., Hempel N. (2017). Insights into the dichotomous regulation of SOD2 in cancer. Antioxidants.

[80] Tonelli C., Chio I.I.C., Tuveson D.A. (2018). Transcriptional Regulation by Nrf2. Antioxid. Redox Signal..

[81] Rani N., Arya D.S. (2020). Chrysin rescues rat myocardium from ischemia-reperfusion injury via PPAR-γ/Nrf2 activation. Eur. J. Pharmacol..

[82] Piantadosi C.A., Carraway M.S., Babiker A., Suliman H.B. (2008). Heme oxygenase-1 regulates cardiac mitochondrial biogenesis via nrf2-mediated transcriptional control of nuclear respiratory factor-1. Circ. Res..

[83] Shen W., Liu K., Tian C., Yang L., Li X., Ren J., Packer L., Cotman C.W., Liu J. (2008). R-α-Lipoic acid and acetyl-L-carnitine complementarily promote mitochondrial biogenesis in murine 3T3-L1 adipocytes. Diabetologia.

[84] Holmström K.M., Baird L., Zhang Y., Hargreaves I., Chalasani A., Land J.M., Stanyer L., Yamamoto M., Dinkova-Kostova A.T., Abramov A.Y. (2013). Nrf2 impacts cellular bioenergetics by controlling substrate availability for mitochondrial respiration. Biol. Open.

[85] Ichimura Y., Waguri S., Sou Y.-s., Kageyama S., Hasegawa J., Ishimura R., Saito T., Yang Y., Kouno T., Fukutomi T. (2013). Phosphorylation of p62 Activates the Keap1-Nrf2 Pathway during Selective Autophagy. Mol. Cell.

[86] Kovac S., Angelova P.R., Holmström K.M., Zhang Y., Dinkova-Kostova A.T., Abramov A.Y. (2014). Nrf2 regulates ROS production by mitochondria and NADPH oxidase. Biochim. Biophys. Acta Gen. Subj..

[87] Ludtmann M.H.R., Angelova P.R., Zhang Y., Abramov A.Y., Dinkova-Kostova A.T. (2014). Nrf2 affects the efficiency of mitochondrial fatty acid oxidation. Biochem. J..

[88] Holmström K.M., Kostov R.V., Dinkova-Kostova A.T. (2016). The multifaceted role of Nrf2 in mitochondrial function. Curr. Opin. Toxicol..

[89] Merry T.L., Ristow M. (2016). Nuclear factor erythroid-derived 2-like 2 (NFE2L2, Nrf2) mediates exercise-induced mitochondrial biogenesis and the anti-oxidant response in mice. J. Physiol..

[90] Lo S.C., Hannink M. (2008). PGAM5 tethers a ternary complex containing Keap1 and Nrf2 to mitochondria. Exp. Cell Res..

[91] Strom J., Xu B., Tian X., Chen Q.M. (2016). Nrf2 protects mitochondrial decay by oxidative stress. FASEB J..

[92] Tsushima M., Liu J., Hirao W., Yamazaki H., Tomita H., Itoh K. (2019). Emerging evidence for crosstalk between Nrf2 and mitochondria in physiological homeostasis and in heart disease. Arch. Pharm. Res..

[93] Ayala A., Muñoz M.F., Argüelles S. (2014). Lipid peroxidation: Production, metabolism, and signaling mechanisms of malondialdehyde and 4-hydroxy-2-nonenal. Oxid. Med. Cell. Longev..

[94] Zhang Y., Sano M., Shinmura K., Tamaki K., Katsumata Y., Matsuhashi T., Morizane S., Ito H., Hishiki T., Endo J. (2010). 4-Hydroxy-2-nonenal protects against cardiac ischemia-reperfusion injury via the Nrf2-dependent pathway. J. Mol. Cell. Cardiol..

[95] Huang Y., Li W., Kong A.N.T. (2012). Anti-oxidative stress regulator NF-E2-related factor 2 mediates the adaptive induction of antioxidant and detoxifying enzymes by lipid peroxidation metabolite 4-hydroxynonenal. Cell Biosci..

[96] Chapple S.J., Cheng X., Mann G.E. (2013). Effects of 4-hydroxynonenal on vascular endothelial and smooth muscle cell redox signaling and function in health and disease. Redox Biol..

[97] López-Bernardo E., Anedda A., Sánchez-Pérez P., Acosta-Iborra B., Cadenas S. (2015). 4-Hydroxynonenal induces Nrf2-mediated UCP3 upregulation in mouse cardiomyocytes. Free Radic. Biol. Med..

[98] Castro J.P., Jung T., Grune T., Siems W. (2017). 4-Hydroxynonenal (HNE) modified proteins in metabolic diseases. Free Radic. Biol. Med..

